# Understanding Adult’s Experiences and Perceptions of How to Maintain Physical Activity: A Systematic Review and Qualitative Synthesis

**DOI:** 10.1007/s12529-024-10335-w

**Published:** 2024-11-18

**Authors:** Claire D. Madigan, Chloe Grimmett, Shane N. Sweet, Amanda J. Daley, Victoria E. Kettle, Bethan Phillips, Henrietta E. Graham

**Affiliations:** 1 Centre for Lifestyle Medicine and Behaviour (CLiMB), Loughborough University, Loughborough, UK; 2 The School of Sport, Exercise and Health Sciences, Loughborough University, Loughborough, UK; 3 School of Health Sciences, University of Southampton, Southampton, UK; 4 Department of Kinesiology and Physical Education, McGill University, Montreal, Canada; 5 Center for Interdisciplinary Research in Rehabilitation of Greater Montreal, Montreal, Canada

**Keywords:** Physical activity, Maintenance, Adults, Qualitative synthesis

## Abstract

**Background:**

Many adults do not meet physical activity recommendations for optimal health, and this is often because people find it difficult to maintain physical activity in the long term. This study focuses on identifying and synthesising factors that may influence the maintenance of physical activity in adults with and without known health conditions.

**Method:**

A systematic review and qualitative synthesis using thematic analysis was conducted. Four databases (MEDLINE, SPORT Discus, APA, and Web of Science) were systematically searched for studies published from inception to February 2023 that included qualitative data about people’s experiences of maintaining physical activity.

**Results:**

A total of 9337 abstracts were screened and 68 studies from 14 countries were included. Six main themes were identified: (1) influence of others (e.g. four forms of social support, accountability); (2) contextual and environmental influences (e.g. cost and access of physical activity, weather); (3) health-related influences (e.g. reflexivity about how physical activity improves health conditions, weight control); (4) making it work (e.g. flexibility, prioritising exercise); (5) habits; and (6) psychological processes (e.g. enjoyment, identifying as a physically active person).

**Conclusion:**

People who maintained their participation in physical activity found it enjoyable, prioritised it, and integrated it into their daily routine. Participants were motivated to continue being physically active when they realised the benefits for their health. Social support, in particular companion support, was a key component facilitating continued engagement. Findings specific to maintenance of physical activity included reflexivity of how physical activity benefited health, flexibility, and identifying as a physically active person.

**Supplementary Information:**

The online version contains supplementary material available at 10.1007/s12529-024-10335-w.

## Introduction

Participation in physical activity has important health benefits [1–4]. To obtain these health benefits, the World Health Organization recommends adults should engage in at least 150–300 min of moderate intensity, or 75–150 min of vigorous intensity, aerobic physical activity each week, or a combination of both. Muscle strengthening activities at least twice per week are also recommended [5, 6].

Despite physical activity being associated with important health benefits, many adults do not achieve recommended levels for optimal health [7–9]. A study that pooled accelerometer data across four European countries found that 72% of adults were not achieving aerobic physical activity recommendations [10]. Another study found only 10–30% of adults participated in strength-based activity each week [11]. Physical inactivity is influenced by several factors, including lack of perceived time, lack of confidence to participate, environmental constraints, and lack of social support [12].

Interventions that focus on helping people initiate physical activity are important for increasing the number of people meeting physical activity recommendations and can be effective [13]. However, helping and encouraging people to maintain this physical activity in the long-term (i.e. physical activity maintenance) are equally important to sustain improvement in health(3).

There is little consensus on the definition of physical activity maintenance [14]. It has been proposed to refer to when physical activity is sustained above a certain threshold for a pre-defined duration (e.g. 150 min of physical activity per week for 6 months), or there are measurable shifts in mechanisms of actions (psychological processes, e.g. self-regulation) that help people to continue to be active [14]. Evidence suggests that physical activity maintenance may be challenging for lots of people [15]. Therefore, understanding how to help people continue with physical activity over the long term is important to ensure that health benefits derived for physical activity are experienced throughout their lives.

Some systematic reviews have examined questions related to developing understanding of the personal characteristics that determine physical activity maintenance, and the processes by which people achieve this goal. Beliefs about capabilities, motivation, and goals have been identified as strong predictors of maintenance [16]. Another review attempted to understand the predictors of lapse (single setback) and relapse (series of relapses) of physical activity maintenance, but was not able to draw firm conclusions due to a lack of data [17]. However, neither of these reviews included qualitative data which can be very important in facilitating understanding at a deeper level of what works to support people to maintain their health behaviours.

### Methods

The systematic review and qualitative synthesis using thematic analysis is registered on PROSPERO: https://www.crd.york.ac.uk/prospero/display_record.php?ID=CRD42021268110 and reported according to the Preferred Reporting Items for Systematic Reviews and Meta-Analyses (PRISMA) [18].

### Study Inclusion Criteria

Qualitative studies with adult participants (≥ 18 years) that reported data about people’s experience of maintaining physical activity were included. As described above, there is no consensus on the definition of physical activity maintenance. As many people take part in physical activity without a dedicated programme (e.g. incidental physical activity) [19], we adopted an inclusive definition. We included studies that recruited participants that were defined as physically active by the study authors or participated in a physical activity programme that specifically explored maintenance. Studies were excluded if the participants were going through an acute health problem/period (e.g. chemotherapy, pregnancy). These studies were excluded because factors that influence physical activity behaviour will differ depending on whether participants are in an acute phase (e.g. pregnancy chemotherapy, waiting for heart surgery) versus after those phases. Studies that focused on initiation of physical activity, and not maintenance, were excluded.

### Search Strategy

Databases were searched from inception to 14 February 2023: MEDLINE, SPORT Discus, APA, and Web of Science. Search terms included physical activity, exercise, follow-up, maintenance, behaviour change, intervention, program, and alternative versions. Searches were limited to adults. Supplementary file 1 provides the search for the MEDLINE database which was adapted for the other databases.

### Study Selection and Data Extraction Process

Search results were uploaded to Covidence systematic review software (Veritas Health Innovation, Melbourne, Australia) [20] and duplicates were removed. Two independent authors (among CM, BP, HG) screened study titles, abstracts, and full texts applying the eligibility criteria. All decisions of inclusion or exclusion were automatically recorded in Covidence, and reviewers were blinded to each other’s decisions. Disagreements were discussed between the two reviewers and resolved by consensus by a third author.

Data about study characteristics were extracted by one author (among BP,CM) and checked by a second (among CM, HG). Information in supplementary Table 1 was extracted from each study, detailing the characteristics, data collection, conceptual methodological framework, analysis, and health condition (if relevant).

### Strategy for Data Synthesis

Reflexive thematic analysis was used to synthesize the findings in NVivo [21] following the methods laid out by Braun and Clarke [22]. Two reviewers (CM, BP) performed line by line coding of the articles (including supplementary files where relevant). All included studies were re-read to ensure that relevant data was captured and appropriately integrated into preliminary codes. Two review authors (CM, HG) reflectively reviewed the codes, and discussed grouping them into themes and sub-themes. Then two further review authors (SS, CG) independently coded a selection of three articles to reflect on whether the themes proposed were interpreted similarly, or if there were further themes that needed to be included. The names of themes and sub-themes were refined. Then the findings were written up. We also refined the themes and sub-themes based on the feedback received from reviewers after submission of the paper.

We considered examining if themes differed by physical activity typology (exercise, structured physical activity, or unstructured/incidental physical activity (e.g. walking)). However, we could not differentiate structured from unstructured as most studies asked about general physical activity which encompasses both. We analysed themes by people with and without known health conditions. We also conducted a sensitivity analysis to consider whether there were any similarities or differences in factors that influenced physical activity maintenance across ethnicities.

### Assessment of Quality

All papers were rated for quality using the Critical Appraisal Skills Programme (CASP) Checklist [23] independently by two authors (from among HG, VEK, CM) and any disagreements were resolved with consensus by a third author (CM). The authors answered 10 questions (using response options of “yes”, “can’t tell”, and “no”) related to the following domains: research aims, relationships between participants and researcher(s), ethical considerations, statement of findings, how valuable the findings were. Finally, the appropriateness was assessed of (1) qualitative methodology, (2) the research design, (3) the recruitment strategy, (4) the data collection, and (5) the data analysis.

## Results

**Fig. 1:**
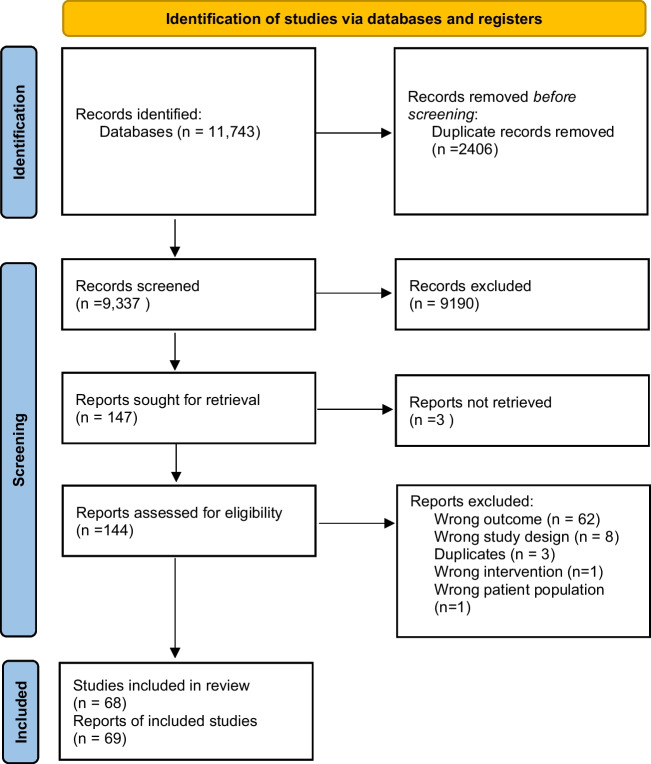
PRISMA flow diagram

The search identified 9337 unique study titles/abstracts after duplicates were removed (Fig. 1). After title and abstract screening, 144 full-text articles were assessed for eligibility. From these, 68 (69 publications — findings from one study published in two manuscripts) studies met the review inclusion criteria.

### Study Characteristics

Supplementary Table 1 details the characteristics of the included studies, including the number (and proportion) of studies by health condition versus no health condition. The total number of participants across the studies was 1651. Most studies were conducted in the USA (*n* = 23) and the UK (*n* = 18). On average, there were 24 participants per study and 55.5% were female. Participants were aged between 18 and 99 years, but only eight studies included people < 40 years [24–31]. Most studies (*n* = 44) did not report the ethnicity of participants. However, there were seven studies that included only people of White ethnicity, seven studies including only people of Black-Afro-Caribbean ethnicity, six studies with over 70% of people of White ethnicity, one study focusing only on people of South Asian ethnicity, and one study of people of Latina ethnicity. Most studies (*n* = 35) used thematic analysis to analyse the qualitative data. Six studies did not state the analysis method used. There were 44 studies that included participants with a specific health condition, the most common being prediabetes/type 2 diabetes (*n* = 14), followed by cardiovascular diseases (*n* = 6) and cancer (*n* = 5).

### Assessment of Quality

Eight studies [24, 32–39] fulfilled the CASP checklist (Supplementary Table 2). The main component not addressed across studies was the relationship between researcher and participant (*n* = 48).

### Synthesis

Six major themes (and 25 sub-themes) were generated as factors that influenced people’s maintenance of physical activity. These were influence of others, contextual and environmental influences, health-related influences, making it work, habits, and psychological factors (Table 1). Supplementary Table 3 details the thematic map with quotes to illustrate findings. Studies that included only people of Black-Afro-Caribbean ethnicity had no quotes in the following sub-themes: goal setting, self-monitoring, physical activity is part of my identity, and comparison to others.

**Table 1:** Number (and proportion) of studies grouped by type of physical activity per theme and subtheme

Theme	Sub-theme	Health condition *N* = 44	No known health condition (*n* = 24)	Total *N* = 68
Influence of others *How others influence a person’s ability to maintain physical activity*	Instrumental social support Gives tangible assistance to take part in physical activity ways, e.g. takes them to the exercise class	*N* = 9 (20%) [28, 36, 40–46]	*N* = 4 (17%) [27, 47–49]	*N* = 13 [27, 28, 36, 40–49]
	Companion social support Completing physical activity with others	*N* = 19 (43%) [28, 32, 33, 40, 41, 43, 46, 50–60]	*N* = 13 (54%) [27, 34, 37, 48, 49, 61–68]	*N* = 32 [27, 28, 32–34, 37, 40, 41, 43, 46, 50–61, 63–68]
	Information social support Gives advice and information about physical activity	*N* = 10 (23%) [32, 38, 40, 41, 44, 53, 54, 69–71]	*N* = 8 (25%) [34, 37, 49, 61, 62, 65, 68, 72]	*N* = 18 [32, 34, 37, 38, 40, 41, 44, 49, 53, 54, 61, 62, 65, 68–72]
	Validation support Providing acceptance or viewpoints that it is good to be physically active	*N* = 13 (30%) [28, 32, 38, 40, 41, 46, 51, 52, 66, 70, 73–75]	*N* = 5 (21%) [47, 61, 65, 66, 72]	*N* = 18 [28, 32, 38, 40, 41, 46, 47, 51, 52, 61, 66, 70, 72–75]
	Accountability *The perceived obligation to be answerable to someone*	*N* = 11 (25%) [38, 40–42, 50–52, 75–78]	*N* = 5 (21%) [34, 39, 49, 64, 79]	*N* = 16 [34, 38–42, 49–52, 64, 75–79]
	Comparison to others *Contrasting the difference and similarities to other people*	*N* = 4 (9%) [32, 40, 54, 56]	*N* = 5 (21%) [34, 39, 61, 62, 66]	*N* = 9 [32, 34, 39, 40, 54, 56, 61, 66]
Contextual and environmental influences *How the physical and social surroundings or conditions in which a person lives influences physical activity maintenance*	Weather *How the weather conditions influence physical activity maintenance*	*N* = 6 (14%) [32, 40, 41, 44, 52, 54]	*N* = 4 (17%) [34, 49, 62, 64]	*N* = 10 [32, 34, 40, 41, 44, 49, 52, 54, 62, 64]
	Cost *The affordability of physical activity to maintain it*	*N* = 8 (18%) [28, 32, 36, 40, 46, 50, 76, 78]	*N* = 2 (8%) [63, 65]	*N* = 10 [28, 32, 36, 40, 46, 50, 63, 65, 76, 78]
	Accessing physical activity *The ease to access physical activity opportunities at home or in the community*	*N* = 19 (43%) [32, 33, 36, 40, 41, 43, 50–52, 54, 57–60, 70, 71, 80–82]	*N* = 4 (17%) [49, 62, 63, 65]	*N* = 23 [32, 33, 36, 40, 41, 43, 49–52, 54, 57–60, 62, 63, 70, 71, 80–82]
	Safety *The perception that it is dangerous to take part in physical activity because of the environment*	*N* = 4 (9%) [32, 33, 55, 75]	*N* = 2 (8%) [49, 72]	*N* = 6 [32, 33, 49, 55, 72, 75]
	Stressful life events *Discrete experiences that disrupt an individual’s usual activities*	*N* = 3 (7%) [42, 75, 76]	*N* = 1 (5%) [49]	*N* = 4 [42, 49, 75, 76]
Health-related influence *The reasons for maintaining physical activity*	Health conditions Managing diagnosable physical health conditions	*N* = 18 (41%) [32, 33, 36, 40, 41, 43, 50, 51, 54, 55, 57–59, 70, 71, 81–83]	*N* = 11 (46%) [27, 35, 37, 47, 56, 61, 62, 64, 65, 68, 83]	*N* = 29 [27, 32, 33, 35–37, 40, 41, 43, 47, 50, 51, 54, 55, 57–59, 61, 62, 64, 65, 68, 69, 71, 81–84]
	Age Maintaining health related to declining health because of age	*N* = 5 (11%) [40, 50, 53, 71, 74]	*N* = 6 (25%) [34, 37, 64, 65, 67, 68]	*N* = 11 [34, 37, 40, 50, 53, 64, 65, 67, 68, 71, 74]
	Physical health *Perceived benefits to physical health*	*N* = 9 (20%) [32, 36, 41, 56, 57, 60, 70, 74, 76]	*N* = 10 (42%) [27, 34, 37, 45, 47, 49, 62, 64, 65, 68]	*N* = 19 [27, 32, 34, 36, 37, 41, 45, 47, 49, 56, 57, 60, 62, 64, 65, 68, 74, 76]
	Maintain mental health *Perceived benefits to mental health*	*N* = 13 (30%) [28, 32, 50, 51, 54, 58, 69–71, 76, 81, 82, 84]	*N* = 11 (46%) [24, 26, 27, 45, 47, 62, 64, 65, 79, 83, 85]	*N* = 24 [24, 26–28, 32, 45, 47, 50, 51, 54, 58, 62, 64, 65, 69–71, 76, 79, 81–85]
	Weight control *Managing and maintaining a body weight*	*N* = 7 (16%) [32, 36, 43, 53, 70, 71, 76]	*N* = 6 (25%) [27, 39, 47, 49, 64, 65]	*N* = 13 [27, 32, 36, 39, 43, 47, 49, 53, 65, 70, 71, 76]
	Health issues *Injuries and illnesses that affect PA maintenance*	*N* = 11 (25%) [32, 38, 40, 41, 44, 46, 51, 57, 58, 66, 80]	*N* = 11 (43%) [37, 39, 45, 47–49, 62–65, 68]	*N* = 22 [32, 37–41, 44–49, 51, 57, 58, 62–65, 68, 80]
Making it work Participants reported using different methods to ensure they continued to maintain their physical activity	Prioritising *The perceived importance of PA*	*N* = 14 (32%) [32, 36, 38, 41, 46, 47, 50, 55, 58, 70, 71, 75–77]	*N* = 11 (46%) [24, 37, 39, 45, 61, 63–65, 68, 79, 85]	*N* = 25 [24, 32, 36–39, 41, 45–47, 50, 55, 58, 61, 62, 65, 68–71, 75–77, 79]
	Goal setting *Setting behavioural targets for physical activity*	*N* = 5 (11%) [36, 41, 58, 69, 81]		*N* = 5 [36, 41, 58, 69, 81]
	Self-monitoring *Recording or measuring their physical activity*	*N* = 6 (14%) [46, 47, 51, 70, 71, 86]	*N* = 3 (13%) [61, 64, 79]	*N* = 9 [46, 47, 51, 61, 64, 70, 71, 79, 86]
	Flexibility Willingness to change or compromise physical activity behaviours	*N* = 9 (20%) [36, 38, 47, 48, 50, 57, 58, 71, 75]	*N* = 8 (33%) [26, 27, 37, 49, 61, 65, 68, 85]	*N* = 17 [26, 27, 36, 37, 47, 48, 50, 57, 58, 61, 65, 68, 71, 75, 85]
Habits *Something that you do often and regularly*		*N* = 14 (32%) [32, 36, 40, 43, 46, 54, 55, 58, 59, 70, 74, 84, 86, 87]	*N* = 11 (46%) [24, 26, 27, 37, 44, 49, 61, 64–66, 68]	*N* = 12 [24, 26, 27, 32, 36, 37, 40, 43, 44, 46, 49, 54, 55, 58, 59, 61, 64, 66, 68, 70, 74, 84, 86, 87]
Psychological factors *Factors related to participants’ thoughts/feelings and opinions*	Physical activity is part of my identity *Perception that your identity is being physically active*	*N* = 4 (9%) [33, 40, 70, 81]	*N* = 3 (8%) [24, 37, 68]	*N* = 7 [24, 33, 37, 40, 68, 70, 81]
	Accomplishment *Feelings of achievement*	*N* = 11 (25%) [32, 46, 56–58, 69, 71, 74, 77, 78, 80]	*N* = 4 (17%) [27, 37, 65, 68]	*N* = 15 [27, 32, 37, 46, 56–58, 65, 68, 69, 71, 74, 77, 78, 80]
	Enjoyment *Physical activity provides pleasure*	*N* = 16 (36%) [32, 33, 36, 40, 41, 50, 54–56, 59, 60, 71, 75, 78, 84, 87]	*N* = 10 (42%) [24, 26, 34, 49, 61–63, 67, 72, 85]	*N* = 26 [24, 26, 32–34, 36, 40, 41, 49, 50, 54–56, 59–63, 71, 75, 78, 85, 87]
	Belief in one’s capability to maintain PA The confidence, competence, perceived behavioural control to keep taking part in physical activity	[36, 38, 40, 46, 52–54, 57, 86]	*N* = 2 (8%)[24, 61]	*N* = 8[24, 36, 38, 40, 46, 52–54, 57, 61, 86]

## Theme 1: Influence of Others

### Social Support

Studies reported different forms of social support that were classified in four ways. The most common form was companion social support (46% of studies) where participants who maintained physical activity reported taking part with others. People liked the social aspect of engaging with physical activity: “When working out, I talk and meet people, and I enjoy that” [32]. This was reported more often by participants with no known health conditions than participants with health conditions (54% versus 41% of studies). Informational social support (26% of studies), e.g. when instructors gave information about physical activity, was recognised as important: “…Someone who knows what you are going through, someone who isn’t going to say do 50 when you can only do 10” [61]. Validation support (26% of studies), when significant others believed physical activity was a good thing, helped them continue to take part. This may be more important for those with a health condition versus those without (30% versus 21% of studies). Instrumental support (19% of studies), i.e., family members/friends giving tangible assistance such as looking after children, helped participants maintain their physical activity.

### Accountability

Participants (25% of studies) reported having someone monitor them was a key part of their continued participation. When monitoring was withdrawn, maintaining physical activity was difficult: “once you’ve left the referral, you don’t have to produce anything to say what you’re doing” [47]. They also felt accountable to friends they exercised with and did not want to “let the other person down”: “Will you be here on Wednesday? … I can’t let them down, I will be here on Wednesday” [69].

### Comparison to Others

Thirteen per cent of studies reported that comparison to others was important to promote maintenance. In seeing others who were physically active increased their motivation to continue: “I was amazed at the number of women in their 80 s that still do pretty strenuous exercise…that’s such a good role model for me to see that and know that I can do it” [62]. Comparison to others was reported more often for adults with no known health condition compared to adults with a health condition (21% versus 9% of studies).

## Theme 2: Contextual and Environmental Influences

### Cost

In 15% of studies, participants reported the cost of physical activity was a barrier to maintaining their physical activity, and adults with a health condition reported it more often than adults without (18% versus 8% of studies). Participants tried to continue, but when the financial costs were too high, they stopped participating:

The exercise center and personnel trainer moved about 1/2 hour from my office making it inconvenient to fit exercise in during the day. I managed to continue the exercises with the personal trainer but after 6 months I found $55.00 per hour too expensive [36].

All the examples provided in the analysis (apart from one) were about the costs of accessing gym facilities/personal trainers. The other example related to being unable to afford a new pedometer.

### Weather

Weather was a barrier to physical activity maintenance (15% of studies), be it too hot or too cold, and was not limited to only outdoor physical activity. One participant mentioned that when it was too hot, they could not go to their aerobics class as there was no air conditioning [32]. However, some participants recognised that they used the weather as an excuse: “Sometimes I will use weather as an excuse…not to exercise. I have a stationary bike…I do have options to work out at home. The weather should not be a factor” [76].

### Access

Ten per cent of studies reported that access influenced maintenance, with some participants reporting they made sure the place they took part in physical activity was close to their home or work as it made them much more likely to keep going. Some participants reported having exercise equipment at home helped them continue to participate. However, if there was a lack of space to store equipment (e.g. bike) or to take part in physical activity in the home (e.g. floor space), it made it more difficult to maintain physical activity:

I live in a house where it’s very crowded and there isn’t much floor space where you get on the floor and really be able to have room to stretch. You’re bumpin’ into furniture here and bumpin’ in there.. .there’s paintings and furniture and there's no place really to do [yoga] [50].

This was more important for those studies that included participants without a known health condition than participants with health conditions (17% versus 8% of studies).

### Safety

Participants (9% of studies) mentioned that safety was an issue, and this was of particular importance to women. It was not only related to how safe they felt exercising in the environment, but also related to safety if they had a known health condition that may make maintaining physical activity more difficult: “Cause it’s always that thing in the back of your mind of, ‘mmm, am I going to push a little bit too hard and have a seizure?’” [33].

### Stressful Life Events

In 6% of studies, stressful live events, such as a death of a family member or job loss, prevented maintenance of physical activity:

Another really hard thing, my husband got sick last year, and our whole lifestyle changed…I wasn’t doing the exercise I should have probably been doing and eating the right foods because I didn’t have my heart in it…it was very, very difficult to cope with [70].

## Theme 3: Health-Related Influences

Throughout this theme, recognising and understanding that their participation in physical activity resulted in personal benefits were important for maintenance.

### Managing Health Conditions

Maintaining physical activity was important to help manage health conditions (43% of studies) and to prevent further deterioration: “I have to do this... or my heart will start falling apart again.” [70].

### Preventing Age-Related Decline

Generally, middle-aged to older participants (16% of studies) spoke about maintaining physical activity to maintain their health as they aged. This was greater in the no known health condition studies than the studies with health conditions (25% versus 11% of studies). They wanted to continue to be able to do things with their families and grandchildren: “I don’t want to be the one in 20 years not being able to get off the couch just because of not maintaining the level [of physical activity] I could” [71].

### Physical Health

As a more general theme, participants reported (particularly more in the no known health condition studies [42%] than health conditions studies [20%]) that physical activity was good for their health, they felt stronger and fitter, and it also made them feel good. They also spoke about preventing lifestyle diseases such as type 2 diabetes and more practical tasks such as being able to put their socks on: “I think that I’m sort of letting myself down when I don’t force myself to be more active. I feel like I’m damaging myself” [32].

### Maintain Mental Health

Forty-six per cent of studies that recruited participants without a known health condition (versus 30% of those studies with a health condition) recognised that continuing to take part in physical activity helped maintain their mental health. They spoke about physical activity being stress relieving, that they felt better after participating and overall, and it improved their mood: “Taking a walk outside can help me get rid of a bad mood. It helps me stop thinking about anything unhappy. … It helps me have a more cheerful mood” [83]. It also helped them reduce their anxiety about health conditions:

Some days I’ve convinced myself that I’m having another heart attack and that today is my last day… I can’t pin down the thing that sparks the negative thoughts that sets that off, but I can pin down the exercise being the thing that kind of zaps you back out of it [51].

### Weight Control

Weight control was reported as a reason for continuing to take part in physical activity as it would help participants to continue managing their weight (16% in health conditions studies versus 25% in no known health conditions studies). However, when weight plateaued some participants reported not seeing the benefits of continuing to be active: “When I first came doing things [PA], I was losing weight, looking better and feeling better but after I had plateaued, what is the reward?" [47].

### Health Issues

There were times when injuries and illnesses reduced participants ability to maintain physical activity, and this was greater in the no known health conditions than the health conditions studies (42% versus 25% of studies).

## Theme 4: Making It Work

### Prioritising

Those who were able to maintain physical activity spoke about prioritising physical activity and making plans to achieve their physical activity (46% in no known health conditions studies versus 32% in health conditions studies): “Well, [my husband] and I both just feel like it’s like going to work. We absolutely have to do that three times a week—it has to be a priority” [63]. In contrast, those who perceived it to be less important were unable to continue with their physical activity:

If somebody turned around to me and said, you’re going to have to walk 10000 steps a day, otherwise you’re going to die in two years’ time, that might do it, but I haven’t got that … I’ve got to do this otherwise it’s a serious thing, because I’m not that unfit [64]. 

### Flexibility

Participants that maintained their physical activity spoke about being flexible (24% of studies) with their physical activity routines to ensure they continued to participate. They recognised that it was okay to deviate from their normal physical activity routine for special occasions and that the important thing was to do some physical activity even if it was not optimal: “It doesn’t have to be an extreme all or nothing kind of participation... because I’ve learned to accept that fact that... it’s something that has to be for life... and there is kind of no such thing as failure” [62].

### Self-monitoring

Participants (14% of studies) reported using self-monitoring, often in the form of pedometers, to record their steps to keep track of their physical activity: “I take my pedometer and there’s not many days I’m not under my 5000 or 10,000 [steps] and, I record it everyday” [37].

### Goal Setting

Some participants (7% of studies) reported that they enjoyed having goals to work towards to help them keep participating in physical activity and this was only mentioned by those with a known health condition.

## Theme 5: Habits

Many participants (46% in no known health condition studies versus 32% with a health condition studies) reported that physical activity was a habit for them and part of their lifestyle that they did not have to consciously think about. This made it easy to continue to maintain it and that they did not think about planning it, that it was just part of their routine: “Exercise is my daily habit, like brushing my teeth or washing my face. It is indispensable” [84]. Participants mentioned that taking part in physical activity daily was a method of maintaining the habit: “I go for a walk every day and never miss a day. This has become a habit. It’s a part of daily activity” [83].

## Theme 6: Psychological Factors

### Belief in One’s Capability to Maintain Physical Activity

Participants mentioned (16% of studies) that because they had started physical activity, they felt more confident about continuing: “Roger and Harriet identified self-efficacy and the confidence that they were able to do a small amount of exercise pushed them to do more” [36]. Participants also stated that they could continue because they were able to start: “One’s become so brainwashed about managing on one’s own. So I was quite clear that I would continue managing on my own” [40]. This suggests that doing small amounts of physical activity initially, then building up the amount over time, builds confidence so that people will continue to be physically active in the long term.

### Physical Activity Is Part of My Identity

Participants (10% of studies) spoke about physical activity as being part of them and what they did — they could not imagine being inactive: “It’s very important to me, it’s really part of my self-concept that fact that I train a lot and I am fit and strong” [26].

### Accomplishment

Some participants (22% of studies) spoke about feeling good that they had achieved their physical activity and that helped them continue to participate: “There is no way I am going to let go of what I have done because I worked too hard” [36]. Those with health conditions mentioned that it was about taking control: “But, you know, if—I think, it’s kind of—it gives you a bit more power, doesn’t it?—your power’s been taken away from you in that treatment time but—and then you want it back. So, you think, so, I’m taking this back.” [64].

### Enjoyment

Thirty-eight per cent of studies mentioned that enjoyment was a reason why people were able to maintain physical activity:

I don’t do it because I have to do it, but I am not like some of my friends who say, look I’ve got to go to walk this morning or I’ve got to go to the gym and swim for half an hour and I’ve got to do my weights and all this type of thing, I do it because I love it [85].

In contrast, some mentioned that they did not like physical activity but overcame this because they recognised the importance of maintaining health and physical function: “I’m in the camp where I hate doing it, but I do it!” [62].

## Discussion

Sixty-eight qualitative studies that explored people’s experience of maintaining physical activity were synthesised. People who maintained physical activity typically found it enjoyable; they prioritised it and had different types of social support to maintain it. Participants that maintained physical activity were able to recognise the benefits that helped improve their health and did not want to lose these benefits. Physical activity became a habit for participants and part of their usual routine. Additional factors that might be unique for maintenance of physical activity (i.e. not reported as important for initiation) included flexibility and identifying as a physically active person.

There were some differences between the studies that focused on people with and without a known health condition. For example, known health issues were less of a barrier for those diagnosed with a health condition. This may be because they have overcome the initial barriers their health condition causes to initiate physical activity. Perceived benefits to physical health and maintaining mental health were reported more frequently among those with no known health condition.

### Comparison to Existing Studies of Physical Activity Maintenance and Differences to Initiation

Participants reported that they identified as a physically active person, and this was important for physical activity maintenance. Caldwell et al. have proposed that identifying as a physically active person is important for sustained behaviour change and suggests this identity transformation will allow people to incorporate new behaviour in their lifestyle and will make them more resilient to disruptions [88].

From a physical environment perspective, cost, access, and weather were reported as factors influencing physical activity maintenance in this review. However, this differs from other studies. For example, Amireault et al. [16] found that access was not associated with physical activity maintenance and Rhodes and Sui [89] articulated that the physical environment is generally stable and so may not be specifically unique to maintenance behaviour. More research is needed to examine the interplay between these constructs and the interaction and the impact they may have on the maintenance of physical activity.

A previous review on physical activity maintenance trials found only seven trials that tested an intervention to help people maintain their activity (after attending a programme) and the effects for physical activity were small (SMD 0.14, 95% CI 0.01 to 0.27) [90]. In contrast, this review consisted of 68 qualitative studies, suggesting we have evidence about factors influencing maintenance and can now apply it to interventions. It is important that guidance for physical activity considers that the initiation and maintenance of physical activity are different behaviours that may be driven by varying factors. The difficulties of getting people to maintain their physical activity might be because of the broad-brush approach we have taken to implementing physical activity behaviours in the population and insufficient time and resources being allocated to considering that initiation and maintenance are not the same behaviours.

### Comparison to Existing Theories of Behaviour Change Maintenance

An overview of theoretical explanations for behaviour change suggests that there are five interconnected themes related to behaviour change maintenance [91]. These are motives (e.g. enjoyment, satisfaction with outcomes, identity), self-regulation, resources (psychological and physical assets), habits, and environmental and social influences [91]. Our review found themes in each of the five themes in the theoretical explanation as well as additional themes. The most prominent themes in this analysis (i.e. with most quotes) were social influences and health-related influences (related to satisfaction with outcomes).

When people realised the benefits of physical activity for their health, they did not want to lose them, and this provided motivation to continue to take part. There was also an example that if only one benefit was experienced from physical activity, for example weight loss, and this was not continually achieved, then participants ceased participating in physical activity. This would suggest that framing the multiple benefits for health that physical activity offers may be important to promoting continued participation. Moreover, it supports the importance of highlighting other factors that promote continued engagement, such as enjoyment, thereby maintaining motivation to continue, even in the absence of recognition of other benefits. This is supported by Grimmett et al.’s conceptual framework of physical activity maintenance in cancer [80]. The authors argue that engagement in physical activity is reinforced by the physical and emotional (i.e. enjoyment) outcomes of participation. They propose that when activity levels are disrupted, re-engaging (and thus maintaining participation) depends on a prioritisation process which will include reflection on all consequences/outcomes of previous engagement. Findings from the current review support this framework.

In past theoretical and empirical articles, social support was reported as a construct important for physical activity maintenance. However, these articles typically pay little attention to the type of social support [89, 91], which limits our understanding of how social support helps with physical activity maintenance. In this paper, we intentionally divided social support data into four types and found companionship was the most common type. However, similar to previous reviews [92, 93], validation support (when significant others believed physical activity was a good thing) and instrumental support (family members/friends giving tangible assistance such as looking after children) were also important for maintaining physical activity. Further, accountability was a key theme in our analysis which is rarely mentioned in the theories of behaviour change. More research may be needed to examine the role of accountability (as its own concept or a form of social support) in physical activity maintenance, especially on identifying how to best foster it [94] in future interventions.

A novel construct identified in this review that is absent from existing behaviour change theories is that of flexibility. People with a flexible approach to physical activity might be able to maintain it as they can cope with lifestyle disruptions that occur. For instance, flexible cognitive restraint has been found to be a mediator of weight loss maintenance whereas rigid restraint is associated with relapse [95].

### Strengths and Limitations

This study included 68 studies and participants (*n* = 1651) from 14 countries, with different health conditions, therefore including a considerable diversity of views and experiences. Themes were similar across studies suggesting consistency of findings. No studies from developing countries were available for inclusion, limiting the replication of the findings in those contexts.

We adopted a pragmatic approach to classifying studies as having recruited participants with a health condition or no known health condition. However, it is possible that some participants did not declare they had a health condition meaning some studies may have been misclassified. We were also unable to delineate between structured exercise and physical activity as most studies asked about physical activity more generally. It could be hypothesised that structured exercise (if there was a clear end to a programme) may be more difficult to maintain as the support ends, but we could not address this question.

Most studies included focused on those who had successfully maintained physical activity with some including those that also had stopped maintaining physical activity which may limit the findings. Only eight studies fulfilled the full CASP checklist, and this was mainly due to unclear reporting. However, it is important to note that many of the included studies were published before the CASP Checklist was developed and will not therefore have benefited from this guidance of how to appropriately report qualitative study methodology. We also chose reflexive thematic analysis as our approach to synthesis but there are other more structured approaches that could have provided more in-depth analysis and could be considered in future [96].

### Recommendations

When encouraging physical activity maintenance, interventions should include components such as self-regulation, social support, habit formation, and helping people reflect on the benefits (satisfaction with outcomes) and helping them to identify as a physically active person. Additionally new components that need further testing are flexibility, creating accountability, and whether teaching prioritisation skills are effective for physical activity maintenance.

## Conclusion

Our findings suggest that some of the common variables reported for physical activity initiation continue to be important for maintenance. These include social support, particularly in the form of companionship, and activities that are enjoyable to people. However, to promote maintenance, interventions may need to focus on unique factors such as reflexivity of the health benefits, flexibility, prioritisation, and helping people identify as a physically active person. When people recognise the multiple benefits to their health that physical activity offers, they are motivated to continue to experience these and consequently strive to maintain a physically active lifestyle.

## Supplementary Information

Below is the link to the electronic supplementary material.

Supplementary file1 (DOCX 276 KB)

## Data Availability

All data relevant to the study are included in the article or uploaded as supplementary information.

## Declarations

### Disclaimer

The views expressed are those of the author(s) and not necessarily those of the NHS, the NIHR, or the Department of Health and Social Care.
