# Supplementary material for: Understanding Adult’s Experiences and Perceptions of How to Maintain Physical Activity: A Systematic Review and Qualitative Synthesis
Source: Int J Behav Med. 2024 Nov 18;32(5):687–701. doi: 10.1007/s12529-024-10335-w (PMC12672732; doi:10.1007/s12529-024-10335-w)
Supplement: Supplementary file 1 — Supplementary file1 (DOCX 276 KB) [file 12529_2024_10335_MOESM1_ESM.docx]

**Supplementary file one: Medline search**

# Searches:

Search: #32 AND #31 AND #30 AND #8 and 2021 or 2022 or 2023  (Publication Years) and Qualitative  (Search within all fields)                                                              Date Run: Tue Feb 14 2023 10:58:15 GMT+0000 (Greenwich Mean Time)                                Results: 222

Search: #32 AND #31 AND #30 AND #8 and 2021 or 2022 or 2023  (Publication Years)                                                       Date Run: Tue Feb 14 2023 10:56:36 GMT+0000 (Greenwich Mean Time)                         Results: 2310

Search: #32 AND #31 AND #30 AND #8                                                   Date Run: Tue Feb 14 2023 10:56:13 GMT+0000 (Greenwich Mean Time)                               Results: 11933

Search: #11 OR #12 OR #13 OR #14 OR #15 OR #16                                                           Date Run: Tue Feb 14 2023 10:56:01 GMT+0000 (Greenwich Mean Time)                        Results: 4385504

Search: #10 OR #17 OR #18                                                          Date Run: Tue Feb 14 2023 10:55:23 GMT+0000 (Greenwich Mean Time)                        Results: 1308215

Search: #7 OR #9 OR #20 OR #21 OR #24 OR #25 OR #23 OR #22 OR #29 OR #28                                                  Date Run: Tue Feb 14 2023 10:54:47 GMT+0000 (Greenwich Mean Time)                   Results: 1381707

Search: (behavi* or lifestyle or life-style) NEAR/5 (chang* or modif*)  (Title)                                                          Date Run: Tue Feb 14 2023 10:52:14 GMT+0000 (Greenwich Mean Time)                   Results: 35197

Search: (behavi* or lifestyle or life-style) NEAR/5 (chang* or modif*)  (Abstract)                                                  Date Run: Tue Feb 14 2023 10:52:08 GMT+0000 (Greenwich Mean Time)                   Results: 209995

Search: (light or moderate or vigorous or vigorous) NEAR/5 activity  (Abstract)                                                     Date Run: Tue Feb 14 2023 10:51:47 GMT+0000 (Greenwich Mean Time)                   Results: 65540

Search: (light or moderate or vigorous or vigorous) NEAR/5 activity  (Title)                                                             Date Run: Tue Feb 14 2023 10:51:14 GMT+0000 (Greenwich Mean Time)                   Results: 10304

Search: (increas* or improv* or higher or chang* or modif*) NEAR/5 exercise or physical activity  (Abstract)                                                          Date Run: Tue Feb 14 2023 10:32:59 GMT+0000 (Greenwich Mean Time)                              Results: 338990

Search: (increas* or improv* or higher or chang* or modif*) NEAR/5 exercise or physical activity  (Title)                                                   Date Run: Tue Feb 14 2023 10:32:44 GMT+0000 (Greenwich Mean Time)                              Results: 95964

Search: (reduc* or decreas* or lower* or chang* or modif*) NEAR/5 (sedentary behav*)  (Title)                                                  Date Run: Tue Feb 14 2023 10:31:20 GMT+0000 (Greenwich Mean Time)                              Results: 557

Search: (reduc* or decreas* or lower* or chang* or modif*) NEAR/5 (sedentary behav*)  (Abstract)                                                          Date Run: Tue Feb 14 2023 10:31:15 GMT+0000 (Greenwich Mean Time)                              Results: 5058

Search: (health or lifestyle or life-style) NEAR/2 behav*  (Abstract)                                                            Date Run: Tue Feb 14 2023 10:30:42 GMT+0000 (Greenwich Mean Time)                          Results: 66166

Search: (health or lifestyle or life-style) NEAR/2 behav*  (Title)                                                    Date Run: Tue Feb 14 2023 10:30:33 GMT+0000 (Greenwich Mean Time)                     Results: 21198

Search: (health or lifestyle or life-style) NEAR2 behav*  (Title)                                                      Date Run: Tue Feb 14 2023 10:30:22 GMT+0000 (Greenwich Mean Time)                     Results: 0

Search: (maintenance or maintain* or extend* or sustain* or persist* or continu*) NEAR/10 (activity or active* or pa or exercis*)  (Title)                                                          Date Run: Tue Feb 14 2023 10:29:54 GMT+0000 (Greenwich Mean Time)                    Results: 16713

Search: (maintenance or maintain* or extend* or sustain* or persist* or continu*) NEAR/10 (activity or active* or pa or exercis*)  (Abstract)                                                  Date Run: Tue Feb 14 2023 10:29:39 GMT+0000 (Greenwich Mean Time)                    Results: 213174

Search: trial  (Abstract)                                                  Date Run: Tue Feb 14 2023 10:28:01 GMT+0000 (Greenwich Mean Time)                    Results: 1133692

Search: trial  (Title)                                                          Date Run: Tue Feb 14 2023 10:27:53 GMT+0000 (Greenwich Mean Time)                    Results: 518966

Search: program or programme  (Title)                                                   Date Run: Tue Feb 14 2023 10:27:37 GMT+0000 (Greenwich Mean Time)                               Results: 508149

Search: program or programme  (Abstract)                                                           Date Run: Tue Feb 14 2023 10:27:32 GMT+0000 (Greenwich Mean Time)                        Results: 1761339

Search: intervention* or preintervention* or pre-intervention* or postintervention* or post-intervention*  (Abstract)                                                                Date Run: Tue Feb 14 2023 10:27:03 GMT+0000 (Greenwich Mean Time)                              Results: 1276177

Search: intervention* or preintervention* or pre-intervention* or postintervention* or post-intervention*  (Title)                                                                Date Run: Tue Feb 14 2023 10:26:26 GMT+0000 (Greenwich Mean Time)                              Results: 272853

Search: "follow up" or maintenance or maintain* or extend* or sustain* or persist* or continu*  (Title)                                                    Date Run: Tue Feb 14 2023 10:26:04 GMT+0000 (Greenwich Mean Time)                              Results: 1119393

Search: risk reduction behavior  (All Fields)                                                           Date Run: Tue Feb 14 2023 10:25:30 GMT+0000 (Greenwich Mean Time)                        Results: 28689

Search: #6 OR #5 OR #4 OR #3 OR #2 OR #1                                                          Date Run: Tue Feb 14 2023 10:25:08 GMT+0000 (Greenwich Mean Time)                        Results: 908025

Search: health behavior  (All Fields)                                                          Date Run: Tue Feb 14 2023 10:24:21 GMT+0000 (Greenwich Mean Time)                               Results: 874838

Search: exercise  (Title)                                                  Date Run: Tue Feb 14 2023 10:23:39 GMT+0000 (Greenwich Mean Time)                    Results: 203617

Search: exercise  (Abstract)                                                         Date Run: Tue Feb 14 2023 10:23:24 GMT+0000 (Greenwich Mean Time)                        Results: 355950

Search: physical activity  (Abstract)                                                          Date Run: Tue Feb 14 2023 10:23:07 GMT+0000 (Greenwich Mean Time)                               Results: 265535

Search: physical activity  (Title)                                                   Date Run: Tue Feb 14 2023 10:22:55 GMT+0000 (Greenwich Mean Time)                        Results: 77203

Search: ALL=(Exercise Therapy)                                                 Date Run: Tue Feb 14 2023 10:22:03 GMT+0000 (Greenwich Mean Time)                        Results: 83404

Search: exercise  (All Fields)                                                         Date Run: Tue Feb 14 2023 10:21:39 GMT+0000 (Greenwich Mean Time)                        Results: 695420

| **Author, year, country, reference**  **Table 2: Characteristics of included studies** | **n** | **% Female** | **Age years (mean/ SD or range)** | **Recruitment setting** | **Ethnicity** | **Data Collection** | **Conceptual Methodological Framework** | **Analysis** | **Health Condition** |
| --- | --- | --- | --- | --- | --- | --- | --- | --- | --- |
| Affuso, 2022, US (29) | 187 | 100% | 41 (12.3) | Online | Black | Survey | Descriptive phenomenological approach | Thematic analysis | NR |
| Alvarado, 2015, Barbados (28) | 17 | 100% | 30 (range 27–34) | Part of a cross sectional survey study | 100% Afro-Caribbean descent | In-depth Interviews | NS | Inductive Thematic analysis | BMI > 25 kg/m2 |
| Austin, 2014, US (72) | 15 | 100% | Range 45-66 years | Posters and presentations through community groups | 100% African American | In-depth Interviews | Physical activity evolution model | Deductive Thematic analysis | NR |
| Bean, 2020, Canada (95) | 14 | 100% | 60 (5.1) | Part of a 3-week diabetes programme | NR | In depth interviews at four time points over a year | Trajectory approach | Deductive-inductive thematic analysis | Pre-diabetes |
| Bethancourt, 2014, US (43) | 52 | 54% | 70.9 (3.7) | Health care provider | 77% white, 13.5% Asian, 7.6% other | 4 x focus groups | Social-ecological framework | Thematic analysis | NR |
| Bilcher-Hansen, international (UK, Canada, Australia, US 2022) (97) | 18 | 44.4% | 41-76 years | Closed online groups for T2DM | NR | Semi structured interviews | M-PAC framework | Thematic analysis | T2DM |
| Billany et al 2022, UK (98) | 13 | 38.5% | 53 (13.0) | Hospital | 92.3% White British | Semi structured interviews | Constructivism paradigm | Framework analysis | Kidney transplant |
| Bjornsdottir, 2012, Iceland (68) | 10 | 100% | 84 (range 72 to 97) | Retirement communities | NR | In depth interviews | Vancouver school of phenomenology | Vancouver school of phenomenology | NR |
| Brunet, 2013, UK (32) | 14 | 100% | 55.3 | Through healthcare providers and referral | 100% Caucasian | Semi-structured in-depth interviews  Questionnaires | NS | Thematic analysis | Breast Cancer |
| Chen, 2014, Taiwan (48) | 18 | 38.9% | 80.6 (7.3) | Recruited through nursing homes | NR | In depth interviews | NS | Content analysis | NR |
| Chong 2022, Australia (99) | 29 | 66% | 74 (6.7) | Recruited through physical activity trial | NR | Semi structured interviews | NR | Inductive thematic analysis | At risk of cognitive decline |
| Collard, 2017, US (33) | 6 | 57.1% | 42 (range 18 – 60) | Epilepsy Action’s Website, newsletter, and support groups – Southwest of England | 90% Caucasian | Focus Groups and semi-structured interviews | Constructionist grounded Theory | NS | Participants with epilepsy |
| Coull 2021, UK (49) | 18 | 28% | 60.5 (range 37 – 73) | Charity health forums, support groups and gymnasium | NS | Semi-structured Interviews | Grounded theory methodology | Inductive Thematic analysis | Myocardial Infarction |
| De Boer, 2022, The Netherlands (30) | 12 | 66.6% | Range 30-99 years | Took part in physical activity programme | NS | Interviews via story telling | COM-B model | Story form | With Chronic disease |
| Deseveaux 2017, Canada, (73) | 11 | 45% | 70.1 (8.5) | Rehabilitation Programs in Toronto – Community | NS | Semi-structured Interviews | NS | Deductive thematic analysis | Older adults with heart failure and COPD |
| Dikareva, 2016, Canada (74) | 12 | 100% | 47 (9.0) | Bariatric clinic | NS | Semi-structured Interviews | NS | Inductive thematic Analysis | Post bariatric surgery |
| Dlugonski, 2012, UK (47) | 22 | 100% | 42.9 (10.2) | Community (database of local residents who had participated in previous research) | NS | Semi-structured Interviews | Social Cognitive Theory | Inductive thematic Analysis | Multiple Sclerosis |
| Dohrn, 2016, US (75) | 18 | 100% | 76.5 (range 66 – 86) | Community | NS | Semi-structured Interviews | NS | Thematic content analysis | Osteoporosis |
| Ekegren, 2020, Australia (71) | 66 | 33% | 50 (15.0) | Recruited as part of a hospital trauma registry | NS | Three structured Interviews over five years | NS | Thematic analysis | Non neurological major trauma |
| Eriksson, 2013, UK (88) | 11 | 45% | Range 61-81 years | Hospital | NS | In-depth semi-structured interviews | Grounded Theory Methodology | Inductive thematic analysis | Parkinson’s Disease |
| Fasczewski, 2018, UK (93) | 15 | 73% | 43.5(10.03) | Using social media | Caucasian | Interviews | Self-determination theory framework | Inductive thematic analysis | Multiple sclerosis |
| Filbay, 2017 UK (54) | 18 | 0% | 51 (11.0) | Recruited from elite cricket players association | NS | Semi-structured interviews | NS | Inductive thematic analysis | None |
| Garland 2009, US (45) | 13 | 84.6% | 56 (4.0) | Community | 84.6% Caucasian | In-depth interviews | Phenomenology | Inductive thematic analysis | Type 2 Diabetes |
| Grant, 2017, UK (80) | 19 | 68% | Range 58-89 | Walk for health group | NS | Interviews | Ethnographic approach | Narrative | All but three had long term condition |
| Grimmett, 2020, UK (76) | 27 | 44% | 66.3 years (range 41 – 79) | PA promotion programmes | NS | Semi-structured interviews | NS | Inductive thematic analysis | Gastrointestinal Cancer |
| Hall, 2001, US (36) | 5 | 40% | 64.8 (range 55 – 82) | Previous Diabetes and research survey | 100% Caucasian | In-depth interviews | NS | Inductive thematic analysis | Type 2 Diabetes |
| Harley, US 2009 (26) | 15 | 100% | Age range 26-45 | African American sorority meetings | 100% African American | In-depth interviews and two focus groups | Grounded theory approach | Grounded theory | NR |
| Harley, US 2014, (82) | 14 | 100% | Median 49 years | Study participants of previous observational study | 100% African American | Semi structured interviews | NS | Thematic analysis | NR |
| Horne, UK 2012 (83) | 46 | 50% | 65.8 | Statutory and voluntary leisure groups and social organisations | 100% South Asian | Focus groups and in-depth interviews | Ethnographic approach | Framework analysis | NR |
| Horne, UK 2013 (69) | 127 | 64% | Age range 60-70 | Field work observation in community settings | 36.2% South Asian and 63.8% European | Focus groups and in-depth interviews | Exploratory approach by naturalistic enquiry | Framework analysis | NR |
| Hubbell, 2020, US (27) | 14 | 100% | Age range 35-65 | Advertisements | 100% African American | Interview | Socioecological model | Content analysis | NR |
| Huberty 2013, US (40) | 30 | 100% | 51.8 (10.2) | Took part in an intervention to increase PA | Caucasian 73.3%  African American 20% | Interview | NR | NR | NR |
| Killingback, 2017, 2021 UK (34,35) | 27 | 81.5% | > 60 years | Community based group exercise programmes | NR | Multiple case study using questionnaires, observation, focus groups and semi-structured interviews. | NR  And Humanisation framework | Thematic analysis | NR |
| Kinnafick, 2006, UK (24) | 12 | 41.7% | range 18-49 | Fitness suite | NR | Semi structured interviews | Grounded theory | NR | NR |
| Kirchoff, 2008, US (44) | 19 | 100% | 40.7 (12.5) | Advertisements in the community | African American100% | Semi structured interviews | Grounded theory | Textual analysis | At risk of diabetes |
| Krczal, 2022, Austria (100) | 35 | 47% | 40-80+ | Convenience sampling | NR | Semi structured interviews | NS | Content analysis | Cardiovascular disease |
| Kuo, 2014, Taiwan (52) | 20 | 55% | 52.3 | Medical centre | NR | Semi structured interviews | Grounded theory | Constant Comparative | Impaired glucose |
| Lee, 2007, US (77,78) | 22 | 59% | 80 | Recruited if completed controlled trial | NS | Interviews | Self-Efficacy Theory | Framework analysis | Older adults with mild to moderate hypertension |
| Loeppenthin, 2014, UK | 16 | 75% | 50 (range 37 – 67) | Rheumatology outpatient clinic | NS | In-depth semi-structured Interviews | Phenomenology | Phenomenological analysis | Rheumatoid arthritis |
| Maula, 2019, UK (101) | 30 | 73.3% | 77.5 (5.7) | Completed strength and balance trial through GP practices | NR | In-depth semi-structured Interviews | NR | Framework analysis | NR |
| Mendoza‐Vasconez, 2022, US (102) | 21 | 100% | 48.29 (9.00) | Individual print intervention and the same intervention technological enhanced | 100% Latina | Semi structured interviews | Transtheoretical model | Thematic analysis framework | NR |
| Midtgaard, 2012, Denmark (50,79) | 23 | 73.9% | 50 | Taking part in a study promoting PA. | NR | Semi structured focus groups | Salutogenetic approach | Descriptive phenomenological approach | Cancer survivors |
| Miller 2017, US (50) | 10 | 68.4% | Aged 65+ | Through community and word of mouth | 100% White | Semi structured interviews | NR | Thematic analysis | NR |
| Nielson, 2014, Denmark (84) | 28 | Men | Middle aged and older | Taken part in intervention study to increase exercise | NR | Focus groups, semi structured | Self-determination theory | NR | NR |
| Peel, 2010, UK (96) | 20 | 45% | 60.8 (range 40 – 80) | GP practices and hospitals | NS | Interviews | NS | NS | T2D patients |
| Penn, 2008, UK (42) | 15 | 46% | 64 (47 – 74) | Community | Caucasian | Semi-structured Interviews | NS | Framework analysis | Impaired glucose tolerance |
| Penn, 2013, UK (42) | 15 | 53.3% | 53.9 | Taking part in a weight management trial | NR | Semi-structured Interviews | Theory domains framework | Framework analysis | T2DM |
| Rise, 2013, US (67) | 23 | 30% | 58 (30 – 72) | Hospital (self-management group education courses) | NS | In-depth semi-structured Interviews | NS | Phenomenological analysis | T2DM |
| Schneider 2022, Switzerland (103) | 19 | 100% | 48.9 (9.7) | Completed structured exercise programme | NS | Semi structured interviews | NS | Thematic analysis | Breast cancer |
| Scott, 2015, US (41) | 32 | 45% | 57.5 ± 13.2 | Exercise referral scheme (community) | Caucasian | Semi-structured Interviews | NS | Framework analysis | Adults with chronic health conditions |
| Seekamp 2015, Australia (92) | 9 | 55.6% | Aged between 40-65 years | Walking trial | NR | Semi-structured Interviews | NR | Thematic analysis | NR |
| Sevild 2020, Norway (25) | 14 | 57.1% | Aged 20-61 years | Healthy life centres (primary care centres focusing on physical and mental health through group experiences | NS | Semi-structured Interviews | Self-determination theory | Systematic text condensation | NR |
| Smith, 2019, Australia (94) | 16 | 88% | 41 (10.9) | Community (advertising method, and from consultant neurologist | NS | Focus-group Interviews | NS | Inductive thematic analysis | Multiple sclerosis |
| Springer, 2005 and 2013, US (37,85) | 12 | 41.7% | 54 | Health/Fitness facility | European American 92% | Semi structured interviews | Grounded theory approach | Grounded theory approach | NR |
| Stewart, 2014, US (53) | 22 | 36.4% | 63.5 (range 45-78) | Pulmonary rehabilitation program | NS | Semi-structured Interviews | Self-determination theory | Content analysis | COPD |
| Swardh, 2008, US (38) | 18 | 77% | 60 (range 34 – 83) | Hospital | NS | Semi-structured Interviews | Phenomenology | Phenomenological analysis | Rheumatoid Arthritis |
| Sweet, 2017, Canada (70) | 15 | 7% | 70 (range 53-79) | Posters and email contact through cardiac rehab programme | NS | Semi-structured Interviews | Theoretical Domains Framework | Content analysis | Patients undergoing cardiac rehabilitation |
| Terranova 2017, Australia (66) | 14 | 100% | 55.6 (8.5) | Completed weight loss intervention after cancer treatment | Caucasian 100% | Semi-structured Interviews | NS | Thematic analysis | Breast Cancer |
| Tulloch, 2013, Canada (65) | 28 | 100% | Age range 39-70 | DARE RCT | NS | Semi-structured Interviews | NS | Inductive thematic themes | T2DM |
| Vela, 2018, US (39) | 22 | 59.1% | Age range 40-65 years | Fitness centres and clinics | NS | Semi-structured Interviews and document review – comparative multiple case study | Social capital, self-efficacy and hedonic theory | Yins five phase analysis | NR |
| [Vilafranca Cartagena](https://sciprofiles.com/profile/2479621), 2022, Spain (104) | 10 | 40% | 58-79 years | Primary Health Centres | NS | Semi structured interviews | NR | Thematic analysis | T2DM |
| Vlcek 2023, Canada (31) | 22 | 63.6% | 19-62 years | Diabetes patient network | NS | Semi structured interviews | NR | Interpretive description analysis | T1DM |
| Wahlich, 2017, UK (51) | 60 | 62% | 70% aged 60-75 years | Physical activity RCTs | White 87% | Semi structured interviews | NS | Thematic analysis | NR |
| Walker, 2018, New Zealand (90) | 5 | 20% | 58 (range 41 – 70) | Rehabilitation program | NS | Semi structured Interviews | Self-determination theory | Inductive and deductive thematic analysis | T2DM |
| Ward 2020, Canada (86) | 9 | 89% | Aged 60+ | Clinical wellness facility providing group physical activity programmes | NS | Semi structured Interviews | Case study model | Inductive thematic analysis | NR |
| Warehime, 2020, US (46) | 22 | 45.5% | 63.41 (9.0) | Part of an RCT | 68.2% Caucasian | Semi-structured- Interviews | Content analysis | Deductive thematic analysis | Heart failure |
| Wycherley, 2012, Australia (91) | 30 | 27% | 56.7 (7.7) | Public Advertisement – community | NS | Semi-structured Interviews | NS | NS | T2DM |
| Yue, 2021, China (87) | 30 | 36.6% | Aged 33-85 years | Hospitals | NS | Semi-structured Interviews | HAPA framework | Template analysis | Coronary heart disease |

**Supplementary Table 2: Assessment of quality using CASP**

| **Study Reference** | **Was there a clear statement of the aims of the research?** | **Is a qualitative methodology appropriate?** | **Was the research design appropriate to address the aims of the research?** | **Was the recruitment strategy appropriate to the aims of the research?** | **Was the data collected in a way that addressed the research ?** | **Has the relationship between researcher and participants been adequately considered?** | **Have ethical issues been taken into consideration?** | **Was the data analysis sufficiently rigorous?** | **Is there a clear statement of findings?** | **How valuable is the research?** |
| --- | --- | --- | --- | --- | --- | --- | --- | --- | --- | --- |
| Affuso, 2022, (29) | yes | yes | can’t tell | Yes | yes | no | yes | can’t tell | yes | yes |
| Alvarado, 2015, (28) | yes | yes | can't tell | yes | yes | yes | yes | yes | yes | yes |
| Austin, 2014, (72) | yes | yes | yes | yes | yes | no | yes | yes | yes | yes |
| Bean, 2020, (95) | yes | yes | can't tell | yes | yes | no | yes | yes | yes | yes |
| Bethancourt, 2014, (43) | yes | yes | yes | yes | yes | no | yes | yes | yes | yes |
| Bjornsdottir, 2012, (68) | yes | yes | yes | yes | yes | no | yes | yes | yes | yes |
| Bilcher-Hansen, 2022 (97) | yes | yes | yes | yes | yes | yes | yes | can’t tell | yes | yes |
| Billany et al 2022, UK (98) | yes | yes | yes | yes | yes | can’t tell | yes | yes | yes | Yes |
| Brunet, 2013, (32,48) | yes | yes | yes | yes | yes | yes | yes | yes | yes | yes |
| Chen, 2014, (48) | yes | yes | yes | yes | yes | no | yes | yes | yes | yes |
| Chong 2022, (99) | yes | yes | yes | yes | yes | can’t tell | yes | yes | yes | yes |
| Collard, 2017 (33) | yes | yes | yes | yes | yes | yes | yes | yes | yes | yes |
| Coull 2021 (49) | yes | yes | yes | yes | yes | no | yes | yes | yes | yes |
| De Boer, 2022 (30) | yes | yes | yes | can’t tell | yes | yes | yes | yes | yes | yes |
| Deseveaux 2017 (73) | yes | yes | yes | yes | yes | no | yes | yes | yes | yes |
| Dikareva, 2016 (74) | yes | yes | yes | yes | yes | no | yes | yes | yes | yes |
| Dlugonski, 2012 (47) | yes | yes | can't tell | yes | yes | no | yes | yes | yes | yes |
| Dohrn, 2016 (75) | yes | yes | yes | yes | yes | no | yes | yes | yes | yes |
| Ekegren, 2020 (71) | yes | yes | can't tell | yes | yes | no | yes | yes | yes | yes |
| Eriksson, 2013 (88) | yes | yes | yes | yes | yes | no | yes | yes | yes | yes |
| Fasczewski,2018 (93) | yes | yes | yes | yes | yes | no | yes | yes | yes | yes |
| Filbay, 2017 (54) | yes | yes | can't tell | yes | yes | no | yes | yes | yes | yes |
| Garland 2009, (45) | yes | yes | yes | yes | yes | can't tell | yes | yes | yes | yes |
| Grant, 2017, (80) | no | yes | yes | yes | yes | yes | yes | no | yes | yes |
| Grimmett, 2020 (76) | yes | yes | can't tell | yes | yes | yes | yes | yes | yes | yes |
| Hall, 2001 (36) | yes | yes | yes | yes | yes | yes | yes | yes | yes | yes |
| Harley,2009 (26) | yes | yes | yes | yes | yes | no | can't tell | yes | yes | yes |
| Harley,2014 (82) | yes | yes | yes | yes | yes | no | yes | yes | yes | yes |
| Horne 2012 (83) | ye | yes | yes | yes | can't tell | no | yes | can't tell | no | yes |
| Horne 2013 (69) | yes | yes | yes | yes | yes | no | yes | yes | yes | yes |
| Hubbell, 2020, (27) | yes | yes | yes | yes | yes | no | yes | yes | yes | yes |
| Huberty 2013, (40) | yes | yes | yes | yes | yes | no | yes | yes | can't tell | yes |
| Killingback, 2017, 2021 (34,35) | yes | yes | yes | yes | yes | yes | yes | yes | yes | yes |
| Kinnafick, 2006, (24) | yes | yes | yes | yes | yes | yes | yes | yes | yes | yes |
| Kirchoff, 2008, (44) | yes | yes | can't tell | yes | yes | no | can't tell | yes | yes | yes |
| Krczal, 2022, (100) | yes | yes | Can’t tell | yes | yes | no | yes | yes | yes | can’t tell |
| Kuo, 2014, (52) | yes | yes | yes | yes | yes | no | yes | yes | yes | yes |
| Lee, 2007 (77) | yes | yes | can't tell | yes | yes | no | yes | can't tell | no | can't tell |
| Loeppenthin, 2014, (78) | yes | yes | yes | yes | yes | no | can't tell | yes | yes | yes |
| Maula, 2019, (101) | yes | yes | yes | yes | yes | no | yes | yes | yes | yes |
| Mendoza‐Vasconez, 2022, (102) | yes | yes | yes | yes | yes | no | yes | yes | yes | yes |
| Midtgaard, 2012 (79) | yes | yes | yes | yes | yes | yes | can't tell | yes | yes | yes |
| Miller 2017 (50) | yes | yes | yes | yes | yes | no | yes | yes | yes | can't tell |
| Nielson, 2014 (84) | yes | yes | yes | yes | yes | no | can't tell | yes | can't tell | yes |
| Peel, 2010, | yes | yes | yes | yes | yes | no | yes | can't tell | can't tell | yes |
| Penn, 2008 (89) | yes | yes | can't tell | yes | yes | no | yes | yes | yes | yes |
| Penn, 2013 (42) | yes | yes | can't tell | yes | yes | no | can't tell | yes | can't tell | yes |
| Rise, 2013, (67) | yes | yes | yes | yes | yes | no | yes | yes | yes | yes |
| Schneider 2022 (103) | yes | yes | yes | yes | yes | no | Can’t tell | yes | yes | yes |
| Scott, 2015 (41) | yes | yes | can't tell | yes | yes | no | yes | yes | yes | yes |
| Seekamp 2016, (92) | yes | yes | yes | yes | yes | no | yes | yes | yes | yes |
| Sevild 2020, (25) | yes | yes | yes | yes | yes | no | yes | yes | yes | yes |
| Smith, 2019, (94) | yes | yes | yes | yes | yes | no | yes | yes | yes | yes |
| Springer, 2005 and 2013, (37,85) | yes | yes | yes | yes | yes | yes | yes | yes | yes | yes |
| Stewart, 2014, (53) | yes | yes | can't tell | can't tell | yes | no | yes | yes | can't tell | yes |
| Swardh, 2008, (38) | yes | yes | yes | yes | yes | yes | yes | yes | yes | yes |
| Sweet, 2017,(70) | yes | yes | yes | yes | yes | no | yes | yes | yes | yes |
| Terranova 2017, (66) | yes | yes | can't tell | yes | yes | no | yes | yes | can't tell | yes |
| Tulloch, 2013, (65) | yes | yes | can't tell | yes | yes | no | yes | yes | can't tell | yes |
| Vela, 2018 (39) | yes | yes | yes | yes | yes | yes | yes | yes | yes | yes |
| [Vilafranca Cartagena](https://sciprofiles.com/profile/2479621), 2022, Spain (104) | yes | yes | yes | yes | yes | no | yes | yes | yes | yes |
| Vlcek 2023, Canada (31) | yes | yes | yes | yes | yes | no | yes | yes | yes | yes |
| Wahlich, 2017, (51) | yes | yes | yes | yes | yes | can't tell | yes | yes | yes | yes |
| Walker, 2018, (90) | yes | yes | yes | yes | yes | can't tell | yes | yes | yes | yes |
| Ward 2020, (86) | yes | yes | yes | yes | yes | no | yes | yes | yes | yes |
| Warehime, 2020 (46) | yes | yes | yes | yes | yes | no | yes | yes | yes | yes |
| Wycherley, 2012, (91) | yes | can’t tell | can't tell | yes | can't tell | no | yes | yes | can't tell | yes |
| Yue, 2021, (81) | yes | yes | yes | yes | yes | yes | yes | yes | yes | yes |

**Supplementary Table 3: Thematic Map**

| Theme | Sub theme | Example quotations |
| --- | --- | --- |
| Influence of others  *How others influence a person’s ability to maintain physical activity* |  |  |
|  | **Instrumental Social Support**  *Gives tangible assistance to take part in physical activity ways e.g., takes them to the exercise class.* | Some women don’t have help - the children have dads that don’t help or other family members so that’s why too. They would have to put the children first. But I have help, luckily. That’s why I decided to start doing something [going to the gym]. (28) |
|  | **Companion Social Support**  *Completing physical activity with others* | “When you have somebody that you're doing it with…We encouraged each other. Both of us liked doing it, so we would get together and exercise whenever we could. It made it easier.” (72) |
|  | **Information Social support**  *Gives advice and information about physical activity* | “I had a trainer for a couple of times and I learned a lot from that. Then I did it on my own and I liked it..” (74) |
|  | **Validation support**  *Providing acceptance or viewpoints that it is good to be physically active.* | ‘‘My husband, he encourages me when I workout. He compliments me, and it makes me feel good and keeps me going.’’ (32) |
|  | **Accountability**  *The perceived obligation to be answerable to someone.* | “I need somebody to keep me going, to keep me on track and make sure that I am not drifting off. I think that was the first year, but it probably rolled on into the second year” (89) |
|  | **Comparison to others**  *Contrasting the difference and similarities to other people* | “I was amazed at the number of women in their 80s that still do pretty strenuous exercise…that’s such a good role model for me to see that and know that I can do it” (43) |
| Contextual and Environmental Influences  *How the physical and social surroundings or conditions in which a person lives influences physical activity maintenance.* | **Weather**  *How the weather conditions influence physical activity maintenance* | ‘‘This summer, I’m just finding it so hot. There is so much humidity that I’m just not able to increase my physical activity at all, and in fact, I’d say, I decreased it.’’(32) |
|  | **Cost**  *The affordability of physical activity to maintain it.* | “One of the reasons I might not be able to go to the gym is because of the costs. Every place is expensive, like $40 a month, and I can’t afford that. I don’t know, I might just have to suffer and exercise by myself at home (in front of) the TV” (82) |
|  | **Accessing physical activity**  *The ease to access physical activity opportunities at home or in the community.* | “[My gym] is really close to where I live. I think that will always be important to me, that it’s close. The closer, the more likely I’ll go” (43) |
|  | **Safety**  *The perception that it is dangerous to take part in physical activity because of the environment.* | “The bears make it really hard . . . . Y’know in Vancouver I don’t think about bears when I go for a walk, and up here in the spring and fall I think about bears, so the bears are a barrier certainly.” (86) |
|  | **Stressful life events**  ***Discrete experiences that disrupt an individual's usual activities.*** | “had a death in my family, one of my favorite uncles. I like went into a depression. So I didn’t come [to the YMCA] for 3 months” (44) |
| Health related influence  *The reasons for maintaining physical activity.* | **Health conditions**  *Managing diagnosable physical health conditions* | “I know that MS is going to take over some day…I’m pushing that out as far as I can by exercising and eating right” (93) |
|  | **Age**  *Maintaining health related to declining health because of age* | “When I look at my father- in- law he’s—he’s—muscle waste. He’s got no strength anywhere. Obviously, arthritis doesn’t help. But, I want to make sure I’ve got the fitness and ability to be able to do more than him when—as I go—go through my older ages.” (76) |
|  | **Physical health**  *Perceived benefits to physical health* | “I use exercise, as reinforcement especially when I don’t feel good. Exercising is part of the equation and because of my knowledge I do it. I fear complications. Maintaining blood glucose levels between 80-110 mg., having more energy, maintaining weight, and mobility of joints give me reinforcement to continue.” (36) |
|  | **Maintain mental health** | “I always feel better. I could be having the worst day and if I choose to do the activity at the end of the day and even if I have done it in the morning, I might get back on the treadmill for 10 minutes or you know, while supper’s in the oven or my husband’s cooking that night or whatever, just kind of get off some steam a little bit and it just makes you feel better.” (47) |
|  | **Weight Control**  *Managing and maintaining a body weight* | “…. and then the older I got, the more I realized I need to stay active, I want to stay active. Number one because you know, otherwise your weight just keeps creeping, creeping up. And what little exercise I do get on a regular basis, it’s hard fighting your weight, at least in my situation, it’s just one of the things I keep active for.” (47) |
|  | **Health issues**  *Injuries and illnesses that affect PA maintenance* | Sometimes I’m just too tired. I find ever since the cancer, I don’t know if it’s the medication or what, but I get tired very easily even just going up a flight of stairs. Not at home but when I go to work I try to walk up the stairs or the escalator and by the time I’m there, I’m out of breath and I’m tired. And I don’t think I used to be that bad before.” (32) |
| Making it work  *Behavioural strategies people use to help them maintain physical activity.* | **Prioritising**  *The perceived importance of PA* | “I prioritize it now. I make a point to make the time to do it, whereas before [cancer], I was like ‘Eh, no biggie, I eat right so it’s no biggie!’ Now it’s like I need it. It’s like a little drug. I need that exercise.” (32) |
|  | **Goal setting**  *Setting behavioural targets for physical activity* | “To me it is paramount to have goals, because I do not find it very fantastic to take those runs alone. I am close to saying that I hate it. No, that may be playing it too strong, but I don't think it is fantastic. I do it, and I do enjoy it afterwards, when it is over. But I would not get it done if it wasn't a prerequisite for signing up for competitions and constantly feel that my form is stable or even improving.” (79) |
|  | **Self-monitoring**  *Recording or measuring their physical activity* | “I take my pedometer and there’s not many days I’m not under my 5000 or 10,000 [steps] and, I record it everyday” (70) |
|  | **Flexibility**  Willingness to change or compromise physical activity behaviours | “It doesn’t have to be an extreme all or nothing kind of participation . . . because I’ve learned to accept that fact that . . . it’s something that has to be for life . . . and there is kind of no such thing as failure. (40) |
| Habits  Something that you do often and regularly. |  | “They’re just part of my daily activities – wake up, pray, meditate, go to gym, go to rec. It’s part of my life. It is actually part of my schedule; at least Monday through Friday, that’s the routine.” (72) |
| Psychological processes | **Belief in one’s capability to maintain PA**  *The confidence, competence, perceived behavioural control to keep taking part in physical activity* | “I came from 1 day a week and then 2 days a week and now I’m coming 4 days a week, they won’t let me come 5 or else I’d be here. And I’m feeling really confident.” (70) |
|  | **Physical activity is part of my identity**  *Perception that your identity is being physically active* | “I have always just felt like there was no other option but to be an active person. I feel like it's a natural part of my life and always has been… I just feel like I just need it to be happy.” (93) |
|  | **Accomplishment**  *Feelings of achievement* | “to keep up with my 5-year-old when she’s running around catching lightening bugs and being able to keep up with her is probably where the sense of accomplishment and positive feeling comes from” (32) |
|  | **Enjoyment**  *Physical activity provides pleasure* | “I do it because I love it. I don’t do it because I have to do it, but I am not like some of my friends who say, look I’ve got to go to walk this morning or I’ve got to go to the gym and swim for half an hour and I’ve got to do my weights and all this type of thing, I do it because I love it.” (54) |

**Supplementary Table 4: PRISMA Cheklist**

| **Section and Topic** | **Item #** | **Checklist item** | **Location where item is reported** |
| --- | --- | --- | --- |
| **TITLE** | | |  |
| Title | 1 | Identify the report as a systematic review. | 1 |
| **ABSTRACT** | | |  |
| Abstract | 2 | See the PRISMA 2020 for Abstracts checklist. | abstract |
| **INTRODUCTION** | | |  |
| Rationale | 3 | Describe the rationale for the review in the context of existing knowledge. | 2 & 3 |
| Objectives | 4 | Provide an explicit statement of the objective(s) or question(s) the review addresses. | 3 |
| **METHODS** | | |  |
| Eligibility criteria | 5 | Specify the inclusion and exclusion criteria for the review and how studies were grouped for the syntheses. | 4 |
| Information sources | 6 | Specify all databases, registers, websites, organisations, reference lists and other sources searched or consulted to identify studies. Specify the date when each source was last searched or consulted. | 4 |
| Search strategy | 7 | Present the full search strategies for all databases, registers and websites, including any filters and limits used. | Supplementary file |
| Selection process | 8 | Specify the methods used to decide whether a study met the inclusion criteria of the review, including how many reviewers screened each record and each report retrieved, whether they worked independently, and if applicable, details of automation tools used in the process. | 5 |
| Data collection process | 9 | Specify the methods used to collect data from reports, including how many reviewers collected data from each report, whether they worked independently, any processes for obtaining or confirming data from study investigators, and if applicable, details of automation tools used in the process. | 5 |
| Data items | 10a | List and define all outcomes for which data were sought. Specify whether all results that were compatible with each outcome domain in each study were sought (e.g., for all measures, time points, analyses), and if not, the methods used to decide which results to collect. | N.A (qual synthesis) |
|  | 10b | List and define all other variables for which data were sought (e.g. participant and intervention characteristics, funding sources). Describe any assumptions made about any missing or unclear information. | 5 |
| Study risk of bias assessment | 11 | Specify the methods used to assess risk of bias in the included studies, including details of the tool(s) used, how many reviewers assessed each study and whether they worked independently, and if applicable, details of automation tools used in the process. | 6 |
| Effect measures | 12 | Specify for each outcome the effect measure(s) (e.g. risk ratio, mean difference) used in the synthesis or presentation of results. | N.A (qual synthesis) |
| Synthesis methods | 13a | Describe the processes used to decide which studies were eligible for each synthesis (e.g. tabulating the study intervention characteristics and comparing against the planned groups for each synthesis (item #5)). | N.A (qual synthesis) |
|  | 13b | Describe any methods required to prepare the data for presentation or synthesis, such as handling of missing summary statistics, or data conversions. | 5 & 6 |
|  | 13c | Describe any methods used to tabulate or visually display results of individual studies and syntheses. | N.A (qual synthesis) |
|  | 13d | Describe any methods used to synthesize results and provide a rationale for the choice(s). If meta-analysis was performed, describe the model(s), method(s) to identify the presence and extent of statistical heterogeneity, and software package(s) used. | 5 & 6 |
|  | 13e | Describe any methods used to explore possible causes of heterogeneity among study results (e.g. subgroup analysis, meta-regression). | N.A (qual synthesis) |
|  | 13f | Describe any sensitivity analyses conducted to assess robustness of the synthesized results. | N.A (qual synthesis) |
| Reporting bias assessment | 14 | Describe any methods used to assess risk of bias due to missing results in a synthesis (arising from reporting biases). | N.A (qual synthesis) |
| Certainty assessment | 15 | Describe any methods used to assess certainty (or confidence) in the body of evidence for an outcome. | N.A (qual synthesis) |
| **RESULTS** | | |  |
| Study selection | 16a | Describe the results of the search and selection process, from the number of records identified in the search to the number of studies included in the review, ideally using a flow diagram. | 7 and figure 1 |
|  | 16b | Cite studies that might appear to meet the inclusion criteria, but which were excluded, and explain why they were excluded. | N.A |
| Study characteristics | 17 | Cite each included study and present its characteristics. | Supplementary file |
| Risk of bias in studies | 18 | Present assessments of risk of bias for each included study. | 7 & supplementary file |
| Results of individual studies | 19 | For all outcomes, present, for each study: (a) summary statistics for each group (where appropriate) and (b) an effect estimate and its precision (e.g. confidence/credible interval), ideally using structured tables or plots. | N.A (qual synthesis) |
| Results of syntheses | 20a | For each synthesis, briefly summarise the characteristics and risk of bias among contributing studies. | N.A (qual synthesis) |
|  | 20b | Present results of all statistical syntheses conducted. If meta-analysis was done, present for each the summary estimate and its precision (e.g. confidence/credible interval) and measures of statistical heterogeneity. If comparing groups, describe the direction of the effect. | 8-16 |
|  | 20c | Present results of all investigations of possible causes of heterogeneity among study results. | N.A (qual synthesis) |
|  | 20d | Present results of all sensitivity analyses conducted to assess the robustness of the synthesized results. | N.A (qual synthesis) |
| Reporting biases | 21 | Present assessments of risk of bias due to missing results (arising from reporting biases) for each synthesis assessed. | N.A (qual synthesis) |
| Certainty of evidence | 22 | Present assessments of certainty (or confidence) in the body of evidence for each outcome assessed. | N.A (qual synthesis) |
| **DISCUSSION** | | |  |
| Discussion | 23a | Provide a general interpretation of the results in the context of other evidence. | 16-19 |
|  | 23b | Discuss any limitations of the evidence included in the review. | 19 |
|  | 23c | Discuss any limitations of the review processes used. | 19 |
|  | 23d | Discuss implications of the results for practice, policy, and future research. | 16-21 |
| **OTHER INFORMATION** | | |  |
| Registration and protocol | 24a | Provide registration information for the review, including register name and registration number, or state that the review was not registered. | 4 |
|  | 24b | Indicate where the review protocol can be accessed, or state that a protocol was not prepared. | 4 |
|  | 24c | Describe and explain any amendments to information provided at registration or in the protocol. | N.A |
| Support | 25 | Describe sources of financial or non-financial support for the review, and the role of the funders or sponsors in the review. |  |
| Competing interests | 26 | Declare any competing interests of review authors. |  |
| Availability of data, code and other materials | 27 | Report which of the following are publicly available and where they can be found: template data collection forms; data extracted from included studies; data used for all analyses; analytic code; any other materials used in the review. |  |
